# The influence of long-term shoulder loading on sagittal spino-pelvic morphology: a population-based retrospective study of Chinese farmers from radiology

**DOI:** 10.1186/s13018-020-01698-3

**Published:** 2020-05-29

**Authors:** Xuyang Zhang, Wei Yang, Zeyu Zheng, Jiasheng Wang, Bao Huang, Shunwu Fan, Xianjun Wang, Fengdong Zhao

**Affiliations:** 1 Department of Orthopaedics, Sir Run Run Shaw Hospital, School of Medicine, Zhejiang University, No. 3, Qingchun Road East, Hangzhou, 310016 People’s Republic of China; 2 Key Laboratory of Musculoskeletal System Degeneration and Regeneration Translational Research of Zhejiang Province, Sir Run Run Shaw Hospital, School of Medicine, Zhejiang University, 3, Qingchun Road East, Hangzhou, 310016 People’s Republic of China; 3 Department of Orthopaedics, Linhai Second People’s Hospital, 198 Dubei Road, Duqiao, Linhai, Taizhou, 317016 People’s Republic of China

**Keywords:** Kyphosis, Weight bear, Shoulder loading, Spine, X-ray

## Abstract

**Background:**

To investigate associations between long-term shoulder loading and sagittal spino-pelvic morphology in Chinese farmers from radiology evidences.

**Methods:**

We retrospectively analyzed 463 back pain patients who attended outpatient and inpatient departments of two hospitals from January 2016 to December 2018, and who had long, standing lateral X-rays according to inclusion and exclusion criteria. One hundred eighty-four of them were farmers with a long history of heavy shoulder loading for over 20 years in their young age, while others were office workers with no reported long-term shoulder loading history. The following parameters were measured by three researchers independently and then analyzed statistically: thoracic kyphosis (TK), lumbar lordosis (LL), thoracolumbar kyphosis (TLK), T9 sagittal offset (T9SO), T1 sagittal offset (T1SO), sacral slope (SS), pelvic incidence (PI), pelvic tilt (PT), C7 tilt (C7T), spino-pelvic angle (SSA), and sagittal vertical axis (SVA).

**Results:**

The “Loading group” included 86 males and 98 females with average age 73.3 (SD 8.3) years, whereas the “Non-loading group” included 126 males and 153 females with average age 63.7 (SD 14.1) years. Age was significantly higher in the loading group (*p* < 0.001), but gender, height, weight, BMI, and BMD were not significantly different (*p* > 0.05). The following spino-pelvic parameters were significantly greater (*p* < 0.05) in the loading group: TK (mean 39.1° vs 32.8°), TLK (25.8° vs 10.1°), and T9SO (12.2° vs 10.1°). Other values were not significantly different between the two groups (*p* > 0.05).

**Conclusion:**

Long-term shoulder loading in youth is a risk factor for pathological thoracic kyphosis especially in the lower thoracic spinal segments when farmers getting older.

## Introduction

Humans are the only fully upright walking vertebrates, and unique anatomical features of the human spine and pelvis have evolved to maintain upright posture and balance [1, 2]. These include the cervical lordosis, thoracic kyphosis, and lumbar lordosis [2]. However, excessive stresses concentrated on the spine can influence these curves and initiate degeneration and deformity [3, 4]. In addition, the pelvis can rotate around the femoral heads, which serve as junctions at which the thoracic-lumbar load is transferred to the lower limbs, and excessive shoulder loading from the spine can result in spino-pelvis imbalance, especially in the sagittal plane [5, 6].

**Fig. 1:**
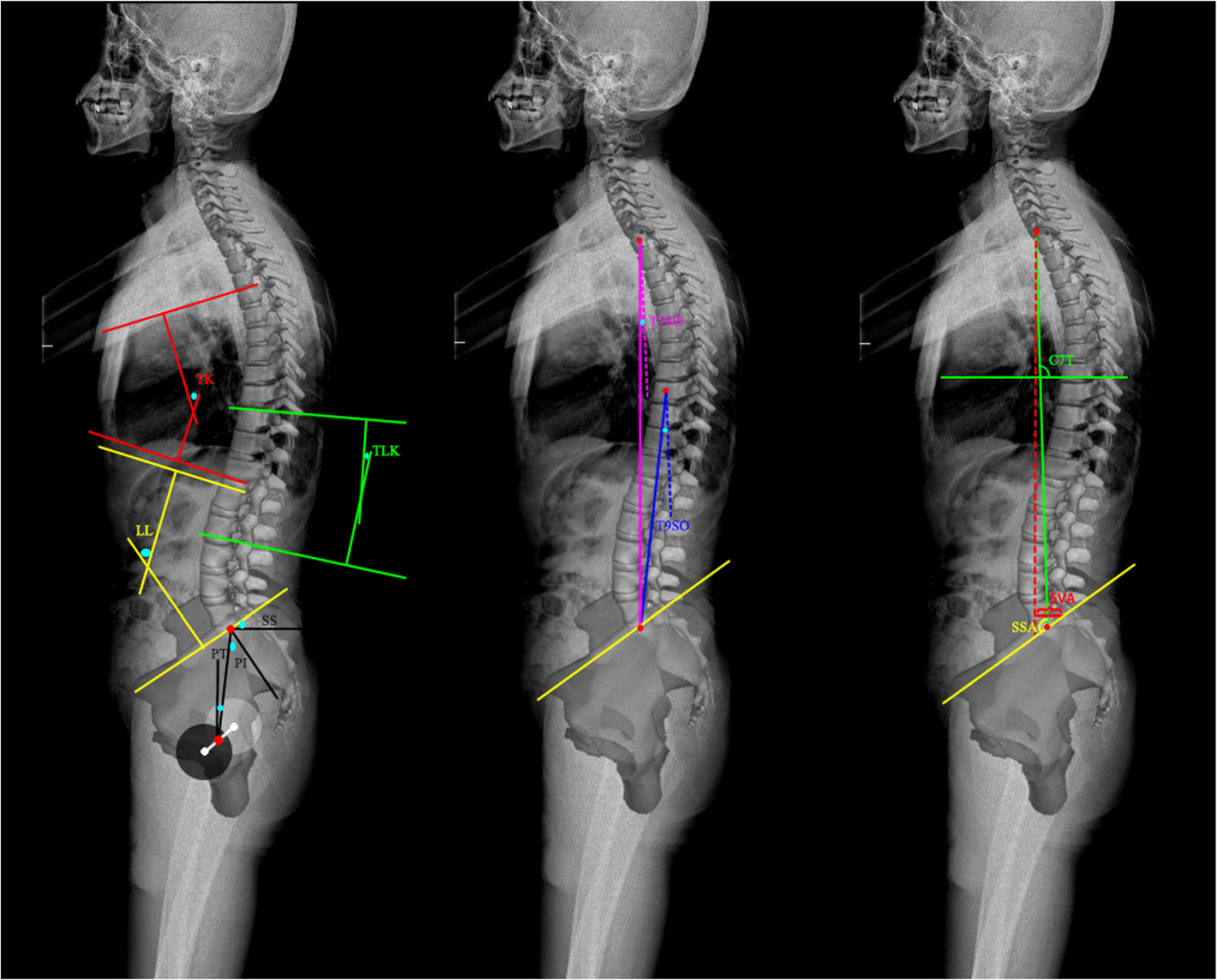
The measuring method of spino-pelvic parameters. TK thoracic kyphosis, LL lumbar lordosis, TLK thoracolumbar kyphosis, T9SO T9 sagittal offset, T1SO T1 sagittal offset, SS sacral slope, PI pelvic incidence, PT pelvic tilt, C7T C7 tilt, SSA spino-pelvic angle, SVA sagittal vertical axis

Spino-pelvic sagittal balance can be assessed by a series of different parameters including thoracic kyphosis (TK), lumbar lordosis (LL), thoracolumbar kyphosis (TLK), T9 sagittal offset (T9SO), T1 sagittal offset (T1SO), sacral slope (SS), pelvic incidence (PI), pelvic tilt (PT), C7 tilt (C7T), spino-pelvic angle (SSA), and sagittal vertical axis (SVA) [6–10]. These are shown in Fig. 1. Several studies have evaluated these spino-pelvic parameters in asymptomatic adults, in European and American Caucasian populations. PI, PT, and SS are similar between females and males, and PI is not related to age, while correlations between other parameters and age are small [11]. Barrey et al. suggested that pelvis shape may influence lumbar degenerative disease and proposed a spinal alignment classification, based on the PI, in normal and pathologic conditions [12]. Vialle et al. measured sagittal radiographs of 300 asymptomatic volunteers and suggested that T9 sagittal offset should be taken into account before surgical treatment because it reflects the sagittal balance of the spine [8]. T9SO is determined by three factors: a linear combination of pelvic incidence, sacral slope, and maximum lumbar lordosis; the thoracic kyphosis; and the pelvic tilt [8]. Recently, Zhu et al. reported sagittal alignment norms in asymptomatic Chinese adults and showed obvious variations between populations with different ethnicity [9]. Several studies presented a strong correlation between SS and the global angle of lordosis. When SS is tilted, the lumbar curvature is high; and contrarily, when SS is rather horizontal, lumbar curvature is flat [12–14].

Little is known, however, about the influence of long-term shoulder physical loading on sagittal parameters, either in Caucasian or Chinese populations. It might be expected that prolonged stooping would increase thoracic kyphosis, for example, or that prolonged upright posture would increase lumbar lordosis, but these possibilities remain to be explored.

China is a traditional agricultural country and farmers occupy the majority of the population. Before the reform and opening up policy of China, many farmers engaged in heavy physical activity from an early age in order to make a living for their whole families, and then kept working until they were too old to continue. With the recent improvement in living standards and development of medical technology, farmers’ life expectancy has been prolonged, and now many farmers seek medical help for spinal deformities and scoliosis. Spino-pelvic degeneration and deformity are very common among farmers, and long-term shoulder loading appears to be an important cause [15]. The present study aims to evaluate the physical condition of the elderly, especially old farmers, and identify any association between long-term shoulder loading and spino-pelvic morphology in the sagittal plane from radiology evidences.

## Methods

### Subjects

The study was reviewed and approved by the institutional review board and the ethics committee of our institution. Patients or their family members agreed to our study and signed the informed consents. We retrospectively reviewed 874 back pain patients who attended outpatient and inpatient departments of two hospitals from January 2016 to December 2018, and who had long, standing lateral X-rays. The inclusion criteria of the subjects were as follows: (1) aged > 50 years, (2) history of back pain > 3 consecutive months, (3) having complete demographic and clinical data, and (4) occupation was farmer or office worker. Exclusion criteria of the subjects were as follows: (1) history of spinal trauma, fracture, tumors, sacroiliac joint diseases or deformity; (2) adolescent idiopathic scoliosis; and (3) history of lumbar spine or pelvis surgery. A total of 463 subjects were reviewed in this study. One hundred eighty-four of them were farmers with a long history of heavy shoulder loading for over 20 years in their young age, while others were office workers with no reported long-term shoulder loading history.

### Clinical data

Demographic and clinical data were reviewed, including age, gender, height, weight, bone mineral density (BMD), and occupation history. All the data were collected by two residents from medical records or by telephone. For radiography, subjects were instructed to stand in a comfortable position, with the hips and knees fully extended [16]. Arms were flexed with the hands resting on supports at the level of their shoulders [16]. All radiographs were obtained in digital format. Parameters related to sagittal alignments were measured three times independently by a post-graduate student, a resident orthopedic surgeon, and a senior orthopedic surgeon, using Image J (Bio-Rad, Hercules, CA, USA). These investigators were blinded to patient information. Each parameter was measured by each of the three researchers, and an average value was calculated. BMD was indicated by a T score.

### Radiographic parameters

The following sagittal parameters of spine and pelvis were evaluated on each long, standing lateral X-ray: thoracic kyphosis (TK), lumbar lordosis (LL), thoracolumbar kyphosis (TLK), T9 sagittal offset (T9SO), T1 sagittal offset (T1SO), sacral slope (SS), pelvic incidence (PI), pelvic tilt (PT), C7 tilt (C7T), spino-pelvic angle (SSA), and sagittal vertical axis (SVA). TK was measured as the angle between the upper end plate of T5 and the lower end plate of T12 (Fig. 1) [17]. LL was measured as the angle between the two lines through the superior end plate of L1 and S1, respectively [17]. TLK was measured as the angle between the upper end plate of T10 and the lower end plate of L2 [17]. For T1SO, we drew a straight line through the midpoint of the T1 vertebral body and the middle point of the line of two femoral heads; then T1SO was defined as the angle between this straight line and the vertical [8]. T9SO was similarly defined in relation to the straight line through the midpoint of the T9 vertebral body [8]. SS was defined as the angle formed between the superior end plate of S1 and the horizontal plane [17]. PT was defined as the angle between the vertical and a straight line joining the centers of the femoral heads and the center of the superior end plate of S1 [17]. PI was defined as the angle between a line drawn from center of the hip axis to the center of the superior end plate of S1 and perpendicular to the end plate [17]. C7T was defined as the angle formed between the horizontal plane and the line joining the center of C7 and the center of the sacral end plate [18]. We drew a straight line between the midpoint of the C7 vertebral body and the midpoint of the S1 superior end plate. And the SSA was defined as the angle between the straight line and the S1 superior end plate [18]. SVA was defined as the vertical distance from the posterior margin of S1 superior end plate to sagittal C7 plumb line [19].

### Statistical analysis

All data were described in the form of the mean value and standard deviation (SD), and analyzed using the SPSS 20.0 software (SPSS Inc., Chicago, IL). Interobserver reliability was analyzed using the kappa statistic. Numerical data were tested by the one-sample Kolmogorov-Smirnov test to determine if it was a normal distribution. The “kyphosis group” was defined as TK > 50° and the rest were the “non-kyphosis group.” Mean values of age, height, weight, BMI, BMD, and sagittal parameters were compared between the two loading groups, using independent-samples *t* tests. Gender and “kyphosis” ratios were compared using the chi-square test. Binary logistic regression was used to identify significant risk factors for kyphosis. Sub-analyses were performed for males and females. Statistical significance was set at a level of *p* < 0.05.

## Results

### Clinical data

All measurement data conform to the normal distribution. The kappa value of was 0.698~0.834 which indicated good interobserver reliability. A total of 212 males and 251 females were enrolled in this study. Average age (SD) was 66.8 (13.3) years, height 161.2 (8.0) centimeters, weight 61.2 (10.4) kilograms, BMI 23.5 (3.2), and BMD 1.11 (0.24). The “Loading group” included 86 males and 98 females with average age 73.3 (SD 8.3) years, whereas the “Non-loading group” included 126 males and 153 females with average age 63.7 (SD 14.1) years. As shown in Table 1, age and the number of patients diagnosed as kyphosis were significantly higher in the loading group (*p* < 0.001), but gender, height, weight, BMI, and BMD were not significantly different (*p* > 0.05). The following spino-pelvic parameters were significantly greater (*p* < 0.05) in the loading group: TK (mean 39.1° vs 32.8°), TLK (25.8° vs 10.1°), and T9SO (12.2° vs 10.1°). Other values were not significantly different between the two groups (*p* > 0.05).

**Table 1:** Comparison of loading and non-loading groups

	Overall			Male			Female		
	Loading	Non-loading	*p* value	Loading	Non-loading	*p* value	Loading	Non-loading	*p* value
Number	184	279		86	126		98	153	
Male/female	86/98	126/153	0.951						
Age	73.3 ± 8.3	63.7 ± 14.1	0.000**	74.3 ± 9.2	63.0 ± 14.1	0.000**	72.5 ± 7.6	64.3 ± 14.1	0.000**
Height	160.8 ± 7.8	161.6 ± 8.2	0.566	167.1 ± 6.2	167.3 ± 6.5	0.892	155.6 ± 4.4	155.7 ± 4.8	0.932
Weight	59.7 ± 11.1	62.6 ± 9.6	0.098	64.6 ± 11.3	66.6 ± 9.1	0.426	55.5 ± 9.3	58.4 ± 8.3	0.168
BMI	23.0 ± 3.3	23.9 ± 3.00	0.075	23.1 ± 3.4	23.8 ± 2.9	0.376	22.9 ± 3.3	24.1 ± 3.2	0.113
BMD	1.05 ± 0.25	1.15 ± 0.24	0.076	1.17 ± 0.21	1.24 ± 0.26	0.471	0.95 ± 0.24	1.09 ± 0.20	0.040*
TK	39.1 ± 17.0	32.8 ± 17.4	0.007**	41.6 ± 16.7	37.3 ± 15.8	0.189	37.2 ± 17.0	29.5 ± 17.8	0.014*
LL	40.5 ± 23.5	37.5 ± 22.0	0.309	44.4 ± 23.9	39.5 ± 18.1	0.232	37.6 ± 23.1	36.0 ± 24.4	0.701
TLK	25.8 ± 16.4	10.7 ± 14.0	0.005**	32.9 ± 9.4	17.1 ± 7.5	0.002**	18.6 ± 19.5	4.1 ± 15.6	0.038*
T9SO	12.2 ± 5.8	10.1 ± 5.2	0.004**	10.9 ± 5.6	9.8 ± 4.3	0.269	13.1 ± 5.8	10.3 ± 5.7	0.006**
T1SO	4.1 ± 5.9	2.9 ± 5.0	0.079	3.2 ± 6.5	1.7 ± 4.1	0.137	4.8 ± 5.4	3.8 ± 5.4	0.271
SS	26.1 ± 12.0	28.4 ± 10.3	0.123	29.2 ± 8.2	29.5 ± 9.2	0.876	23.8 ± 13.8	27.5 ± 11.0	0.079
PI	46.1 ± 11.7	46.7 ± 12.2	0.706	46.6 ± 10.6	46.0 ± 11.3	0.781	45.7 ± 12.6	47.2 ± 12.9	0.501
PT	22.0 ± 12.3	19.4 ± 13.3	0.123	19.5 ± 11.1	17.4 ± 10.2	0.333	24.0 ± 13.0	20.8 ± 15.1	0.218
C7T	93.2 ± 8.1	92.4 ± 6.6	0.402	92.4 ± 6.3	93.4 ± 6.0	0.446	93.8 ± 9.2	91.7 ± 6.9	0.125
SSA	113.4 ± 14.5	116.1 ± 14.2	0.152	116.2 ± 10.9	116.2 ± 11.5	0.982	111.3 ± 16.5	116.1 ± 15.9	0.091
SVA	41.2 ± 59.0	32.2 ± 59.2	0.247	39.0 ± 62.9	42.1 ± 56.8	0.794	42.9 ± 56.3	24.8 ± 60.2	0.081
Kyphosis Y/N	77/107	66/213	0.002**	38/48	33/93	0.046*	39/59	33/120	0.019*

*TK* thoracic kyphosis, *LL* lumbar lordosis, *TLK* thoracolumbar kyphosis, *T9SO* T9 sagittal offset, *T1SO* T1 sagittal offset, *SS* sacral slope, *PI* pelvic incidence, *PT* pelvic tilt, *C7T* C7 tilt, *SSA* spino-pelvic angle, *SVA* sagittal vertical axis

Values are mean ± SD

*Indicates *p* value < 0.05, ** indicates *p* value < 0.01

### Stratified statistics by gender

In males, the mean age was significantly higher in the loading group than in the non-loading group, but of all the spino-pelvic parameters, only TK was significant higher in the loading group (Table 1). In females, age was also higher in the loading group, as were the following spino-pelvic parameters: TK, TLK, and T9SO. What is more, BMD in females was also significantly lower in the loading group which suggested that BMD might influence the spino-pelvic morphology in female group. The proportion of patients with “kyphosis” (TK > 50°) was significantly higher in the loading than non-loading groups, suggesting shoulder loading might be a risk factor for kyphosis.

### Analysis of kyphosis factors

Mean age was significantly higher in the “Kyphosis” than “Non-kyphosis” group (60.7 vs 56.1 years) as shown in Table 2, but other factors such as height, weight, BMI, and BMD were not significantly different. Bivariate logistic regression analysis of kyphosis diagnosis and shoulder loading suggested that shoulder loading could be an independent risk factor of kyphosis (Table 3), adjusted for age.

**Table 2:** Differences between the “Non-kyphosis” and “Kyphosis” groups

	Non-kyphosis	Kyphosis	*p* value
Age (years)	56.1 ± 13.7	60.7 ± 9.4	0.049*
Height	161.7 ± 8.1	158.1 ± 6.4	0.063
Weight	61.7 ± 10.5	58.3 ± 9.5	0.183
BMI	23.5 ± 3.2	23.4 ± 3.9	0.866
BMD	1.12 ± 0.24	1.05 ± 0.31	0.440

Values are mean ± SD

*Indicates *p* value < 0.05

**Table 3:** Bivariate logistic regression analyses to predict “Kyphosis” diagnosis from “Loading”

Adjustment	Odds ratio	95% confidence interval	*p* value
Model 1	3.176	1.573–6.413	0.001**
Model 2	2.774	1.321–5.825	0.007**
Model 3	6.123	1.830–20.489	0.003**
Model 4	5.600	1.680–18.665	0.005**
Model 5	6.160	1.822–20.827	0.003**

Model 1, unadjusted (“Loading” was the only predictor variable). Model 2, adjusted for age; model 3, adjusted for age and height; model 4, adjusted for age and weight; model 5, adjusted for age, height, and weight

*Indicates *p* value < 0.05, ** indicates *p* value < 0.01

## Discussion

Degenerative spinal kyphosis in the elderly is a common phenomenon, with the age increasing and the stability of spine decreasing [20]. Recently, there has been an increasing emphasis on the understanding of sagittal spinal alignment and many factors can influence the spino-pelvic morphology, such as age, BMD, and occupation [6–10, 21]. Therefore, an excessive stress concentrated on the spine may influence the physiological curvature and bring degeneration and deformity of spine [3, 4]. In China, as a developing country, there is still a large group of old farmers who engaged in heavy shoulder loading work when they were young. Whether the shoulder loading in young will aggravate the spine deformity as they get older is worth investigating to prevent the spine deformity.

### Summary of results

Our study compared 184 farmers, who subjected their backs to > 20 years of heavy shoulder loading, with 279 office workers who did little occupational shoulder loading. Measures of thoracolumbar kyphosis were significantly higher in the “Loading” group than in the “Non-loading” group. What is more, the effect of heavy shoulder loading on kyphosis is more notable in females than males because of the low estrogen level.

### Relationship to previous study

The deformation of bone subjected to loads is not instantaneous but varies with time [22]. As suggested by Luo, deformity of vertebrae arises from an accelerated “creep” mechanism [23, 24], and vertebroplasty could reduce the progressive creep deformity of fracture [25]. Meanwhile, O’Callaghan suggested that with the increase of vertebral trabecular bone damage substantial creep deformation may occur even when the vertebra was under physiological loads [26]. Therefore, vertebrae kyphotic posture of Chinese farmers leads to anterior wedge deformity as a result of (a) fractures and (b) bone creep attributed to long-time shoulder loading.

### Explanation of results

The strength of the lumbar spine is higher than that of the thoracic spine, and in the lumbar region, it is also strengthened by the paravertebral structure such as muscle [27]. Therefore, thoracic spine could bear less stress than lumbar spine, and when excessive stress concentrated on the shoulders, the stress transferred from shoulder to thoracic vertebrae [28, 29]. If the stress is beyond the range of the spine bearing, it could lead to wedge change, instability, and cumulative injury in thoracic spine, and cause deformity at last [29]. In loading group, the paravertebral muscle may be much stronger for their daily exercise by heavy shoulder loading [30]. So the lumbar spine does not show significant difference in the morphology as well as the non-loading group. On the other hand, as the development of economic level and improvement of medical conditions, more and more patients seek for medical help when the symptom appears rather than bear for a long time until the severe deformity of spine or pelvis. As for the effects of nutrition and economic conditions on spinal morphology, since the data are collected in the same province, we assume the economic conditions and nutritional status of the subjects are at a close level. Meanwhile, nutritional status may also be reflected from BMI to some extent. It is certain that back pain will affect the stand position, especially the severe back pain. However, 3 months of pain may not cause significant changes in spinal morphology. When there is pain in both groups, the difference between the two groups may not be significant affected by the pain.

### Weaknesses of this study

Patients were all recruited in Zhejiang province which is a developed region in China, and the natural progression of their spinal curvature may have been interrupted by recent medical and social changes. Meanwhile, the patient data came from only two hospitals and the sample size was barely adequate. Also, statistical results may have been biased by missing data, such as BMD. And due to relatively limited number of cases, it is difficult to analyze the relationship between concrete loading time and sagittal spino-pelvic morphology. Our next research plans to choose more hospital to increase the sample size and investigate the less developed provinces in central and western China. Since office works have history of long-period sitting, which could also affect the spine morphology, they may not be the best control group. Compared with long-time shoulder loading on the spine, the loads carried by the office workers are not nearly as great. However, this may still be a limitation of our study. What is more, with the increase of age, disc degeneration will inevitably occur in both groups of subjects, which may be hard to exclude.

## Conclusions

Long-term shoulder loading in youth is a risk factor for pathological thoracic kyphosis especially in the lower thoracic spinal segments when farmers getting older.

## Data Availability

The datasets used and analyzed during the current study are available from the corresponding author on reasonable request.

## Abbreviations

TK: Thoracic kyphosis
LL: Lumbar lordosis
TLK: Thoracolumbar kyphosis
T9SO: T9 sagittal offset
T1SO: T1 sagittal offset
SS: Sacral slope
PI: Pelvic incidence
PT: Pelvic tilt
C7T: C7 tilt
SSA: Spino-pelvic angle
SVA: Sagittal vertical axis
BMD: Bone mineral density
BMI: Body mass index
